# Development of a mechanistic model to predict synthetic biotic activity in healthy volunteers and patients with phenylketonuria

**DOI:** 10.1038/s42003-021-02183-1

**Published:** 2021-07-22

**Authors:** Mark R. Charbonneau, William S. Denney, Nicholas G. Horvath, Pasquale Cantarella, Mary J. Castillo, Marja K. Puurunen, Aoife M. Brennan

**Affiliations:** 1 Synlogic, Inc., Cambridge, MA USA; 2 Human Predictions, LLC, Cambridge, MA USA

**Keywords:** Computational models, Pharmacology

## Abstract

The development of therapeutics depends on predictions of clinical activity from pre-clinical data. We have previously described SYNB1618, an engineered bacterial therapeutic (synthetic biotic) for the treatment of Phenylketonuria (PKU), a rare genetic disease that leads to accumulation of plasma phenylalanine (Phe) and severe neurological complications. SYNB1618 consumes Phe in preclinical models, healthy human volunteers, and PKU patients. However, it remains unclear to what extent Phe consumption by SYNB1618 in the gastrointestinal tract lowers plasma Phe levels in PKU patients. Here, we construct a mechanistic model that predicts SYNB1618 function in non-human primates and healthy subjects by combining in vitro simulations and prior knowledge of human physiology. In addition, we extend a model of plasma Phe kinetics in PKU patients, in order to estimate plasma Phe lowering by SYNB1618. This approach provides a framework that can be used more broadly to define the therapeutic potential of synthetic biotics.

## Abstract (web summary)

Mark Charbonneau et al. develop a mathematical model that describes the behavior of an engineered bacterial therapeutic, SYNB1618, in the human gastrointestinal tract and its effects on plasma phenylalanine (Phe) levels. This model enables the authors to predict plasma Phe lowering by SYNB1618 in patients with phenylketonuria.

## Introduction

Evidence is accumulating that live biotherapeutics comprised of engineered microbes (synthetic biotics) can be used to address the mechanisms of human diseases^1^. The tools of synthetic biology enable rapid and cost-effective development of synthetic biotic prototypes^1,2^. However, the clinical development of therapeutics requires compliance with strict regulatory guidelines and does not scale similarly. Therefore, it is essential to develop testing strategies that can be applied early in the development process to characterize the function of engineered strains, optimize potency, and establish high confidence in their translational potential. For synthetic biotic medicines, environmental conditions are important determinants of strain viability and metabolism, and methods that simulate the conditions of the human gastrointestinal tract (GI), such as the simulated human intestinal microbial ecosystem, are useful for characterizing the viability and function of engineered strains^3^. However, these simplified in vitro simulations lack host cells and tissue architecture. By contrast, animal models may be used to study synthetic biotic function in the context of the complete host organism, but the translational value of animal models varies by species and genotype. For example, animal models differ from humans in some physiological respects, including gastric pH, transit times, and gut microbiota composition^4,5^. In addition, relevant disease models may not be available or cost-effective in model organisms.

From the perspective of engineered bacterial cells, the host environment represents a complex, dynamic system. In the case of oral administration of a synthetic biotic, strain activity is a function of various processes, including gastric emptying, changing intestinal pH, and dose. Predicting the translational value of engineered strains thus necessitates a move toward mathematical frameworks for integrating data from in vitro and in vivo model systems to predict behavior in these dynamic conditions. Mechanistic modeling approaches have been widely used to accelerate drug development for other modalities, including small molecules and recombinant proteins, by modeling the pharmacokinetic and pharmacodynamic properties of drug candidates across a wide assortment of therapeutic indications^6^.

Phenylketonuria (PKU) is a rare genetic disease that results in reduced activity or complete elimination of the enzyme, phenylalanine (Phe) hydroxylase, which converts the essential amino acid, Phe, to tyrosine^7^. For patients with PKU, dietary protein consumption causes prolonged elevation of plasma Phe concentrations and can lead to severe cognitive impairment in infancy if left untreated, among other sequelae^7^. In addition to disrupted Phe metabolism, recent work performed using non-targeted metabolomics has also revealed a set of disrupted urinary metabolites in untreated PKU patients, though the clinical relevance of these compounds remains undetermined^8^.

Current treatment for patients with PKU includes life-long stringent restriction of dietary Phe, supplemented with low-Phe amino acid and trace element mixtures^9^. However, adherence to the PKU diet regimen commonly decreases with age, and some children and most adults with PKU continue to have Phe levels above the recommended range^10,11^. Two pharmacologic treatments currently exist for PKU. A subset of patients with PKU responds to treatment with sapropterin dihydrochloride, a stable analog of the tetrahydrobiopterin cofactor of phenylalanine hydroxylase (PAH)^12^. Pegvaliase-pqpz, a second therapeutic approach for PKU, is composed of a recombinant enzyme, phenylalanine ammonia lyase (PAL), conjugated to *N*-hydroxysuccinimide-methoxypolyethylene glycol and is administered daily by subcutaneous injection. Pegvaliase-pqpz is associated with potentially severe allergic reactions, and its use is currently approved only for adult PKU patients who have uncontrolled blood Phe concentrations >600 μmol/L on existing management^13^. There remains a need for an oral treatment that could be used by all PKU patients regardless of age or genetic background.

We have recently described the development of a synthetic biotic strain of *E. coli* Nissle 1917, called SYNB1618, designed to consume Phe in the human GI^14^ (Supplementary Figure 1). SYNB1618 degrades Phe by the expression of two distinct mechanisms: (1) the conversion of Phe to *trans*-cinnamic acid (TCA) by the enzyme PAL, and (2) the conversion of Phe to phenylpyruvic acid (PPA) by the enzyme l-amino acid deaminase (LAAD). A high-affinity Phe transporter, PheP, increases the influx of substrate into the cell. For biocontainment, the *dapA* gene, encoding 4-hydroxy-tetrahydropicolinate synthase was deleted. This deletion renders SYNB1618 unable to divide without added diaminopimelic acid, a component of the bacterial cell wall, in the environment. TCA produced by PAL is released from the cell, absorbed in the GI, converted in the liver to hippuric acid (HA), and ultimately excreted in the urine. Urinary HA can also be derived from dietary sources, including flavonoids found in fruits and vegetables^15^. However, the use of controlled diets can limit dietary contributions to urinary HA excretion, rendering HA an informative urinary biomarker for SYNB1618 PAL activity in vivo^16^. We have previously shown that oral administration of SYNB1618 significantly lowered blood Phe concentrations in a mouse model of PKU and resulted in dose-dependent production of the urinary biomarker, HA, in healthy non-human primates (NHP)^14^. A recent first-in-human study in healthy volunteers and PKU patients demonstrated that SYNB1618 was generally well tolerated^16^. This study also revealed a dose-dependent production of the strain-specific biomarkers, TCA and HA, upon administration of SYNB1618, confirming Phe consumption by the expressed PAL enzyme in humans. However, it remains to be determined how Phe consumption by SYNB1618 in the GI impacts plasma Phe concentrations in patients with PKU.

Herein, we describe the construction and validation of mechanistic models of SYNB1618 PAL activity in the dynamic conditions of the human upper GI, as well as the effects of dietary Phe removal on plasma Phe concentrations in PKU patients. We show that a model of Phe consumption by SYNB1618 accurately describes biomarker production observed in GI simulation assays, in NHPs, and in healthy human subjects. In addition, we demonstrate that our extension to a previously described model of plasma Phe kinetics to include the effects of dietary Phe accurately captures plasma Phe data from healthy subjects after oral administration of Phe. Lastly, we combine these two models to obtain estimates of plasma Phe lowering in PKU patients that suggest it is feasible to achieve >20% plasma Phe lowering with SYNB1618 in PKU patients.

## Results

### Modeling SYNB1618 PAL activity in vitro

We first aimed to develop mathematical representations of Phe consumption by the synthetic biotic strain, SYNB1618, that could describe strain activity in vitro and in vivo. SYNB1618 consumes Phe via two independent mechanisms (PAL and LAAD)^14^. Phe consumed by the PAL enzyme is converted to TCA, which in turn is converted to HA in vivo. HA can be detected in urine and the change of urinary HA concentration from baseline serves as a quantitative biomarker of PAL activity^14^. Urinary HA data can thus be used to validate predictions of in vivo strain activity. By contrast, the product of the LAAD enzymatic reaction, PPA, is not a suitable biomarker for strain activity in vivo, as oral administration of PPA does not lead to increases in urinary or plasma phenylpyruvate^14^. This may be attributable to the low bioavailability of this compound or the conversion of PPA to other metabolites by the host and/or endogenous microbiota. In vivo activity of the LAAD enzyme has been demonstrated qualitatively by detection of the metabolic end-product phenyllactic acid (PLA) in urine samples from healthy subjects dosed with SYNB1618^16^. However, this observation did not achieve statistical significance, possibly due to the production of PLA through other mammalian pathways and substantial background concentrations of PLA in PKU patients. Together, the current data regarding LAAD activity in vivo are insufficient to support validation of SYNB1618 LAAD enzyme activity. As such, we have focused our efforts on modeling the PAL mechanism of Phe consumption by SYNB1618.

**Fig. 1:**
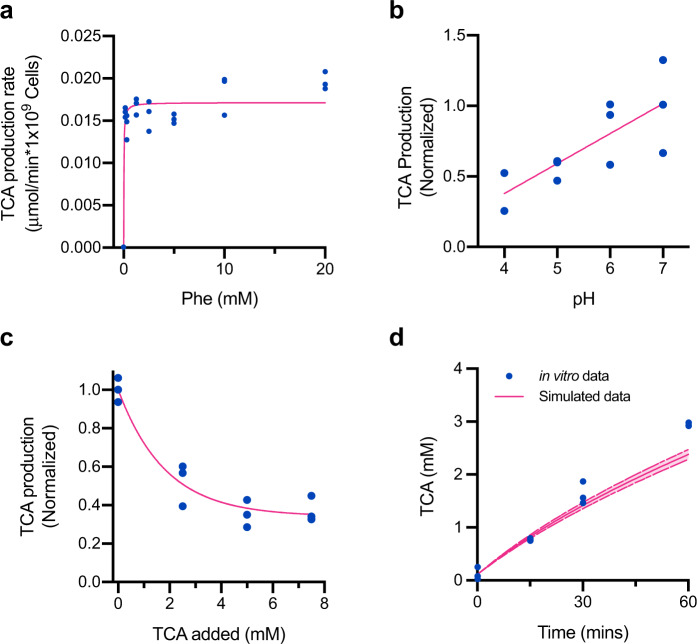
Model of SYNB1618 PAL activity in vitro. **a** TCA production rate by SYNB1618 in PBS containing various initial concentrations of Phe. Points represent experimental replicates (blue; *n* = 3 replicate cultures per initial Phe concentration), with rates normalized to SYNB1618 cell density and time. The Michaelis–Menten curve fit to the experimental data is shown in pink (*R*^2^ = 0.7805). **b** Effect of pH on TCA production by SYNB1618 at 20 mM Phe. Points represent experimental replicates (blue; *n* = 3 replicate cultures per treatment) and indicate the fraction of TCA produced at pH 7.0. Linear regression to the data is shown in pink (*R*^2^ = 0.6002). **c** Effect of exogenous TCA on de novo d5-TCA production by SYNB1618 incubated with 20 mM d5-Phe. Points represent experimental replicates (blue; *n* = 3 replicate cultures per treatment) and indicate the fraction of d5-TCA produced de novo in the absence of exogenously added unlabeled TCA. An exponential decay curve fit to the data is shown in pink (*R*^2^ = 0.9361). Comparison of TCA production by SYNB1618 in an in vitro gastric simulation (IVS) assay (blue) and simulated TCA production using the SYNB1618 in vitro PAL activity model (solid pink line). Points represent experimental replicates (*n* = 3 replicate measurements per time point), and upper and lower bounds for simulated data (dashed pink lines) represent simulations performed using 95% confidence intervals for the PAL activity parameters, *K*m_PAL_ and *V*max_PAL_, as described in the main text.

SYNB1618 PAL activity was represented in the model as an irreversible reaction following Michaelis–Menten kinetics that produces TCA (See Methods; Eqs. 1–2). PAL is a multimeric cytosolic enzyme that requires the transport of Phe into the cell via the Phe-specific transporter, PheP (Supplementary Figure 1). For this reason, as well as due to uncertainty regarding the abundance of enzymatically active PAL in the cytosol, the kinetic parameters of Phe consumption by the PAL pathway (*K*m_PAL_ and *V*max_PAL_) were estimated in a whole-cell context by measuring the rate of TCA production within 1 hour across a range of Phe concentrations in vitro, using SYNB1618 prepared without LAAD induction (*n* = 3 replicates for each Phe concentration; Fig. 1a). Luminal pH varies throughout the human stomach and small intestine, so we also examined the effects of pH on TCA production by SYNB1618 in vitro and discovered a linear relationship between environmental pH and PAL activity at a fixed concentration of 20 mM Phe (*n* = 3 replicates per pH tested; Fig. 1b). Inhibition of PAL activity by pH was represented in the model using an inhibition term (*K*i_pH_). In addition, we have observed that exogenous TCA can inhibit PAL activity. To determine the effect of exogenous TCA on PAL activity in a whole-cell context, SYNB1618 was incubated with increasing concentrations of TCA and provided isotopically labeled Phe (d5-Phe) as a substrate. The rate of d5-TCA production over one hour was measured by liquid chromatography-tandem mass spectrometry (LC-MS/MS), revealing that increasing concentrations of extracellular TCA can decrease de novo d5-TCA production by up to 60% (*n* = 3 replicates per level of exogenous TCA; Fig. 1c). Inhibition of PAL activity by extracellular TCA concentration was also represented in the model using an inhibition constant (*K*i_TCA_; see Methods).

To simulate key aspects of oral dosing in humans, including luminal oxygen concentration, gastric pH, and pepsin activity, as well as to validate our model of SYNB1618 PAL activity, we designed an in vitro gastric simulation (IVS) assay (see Methods). IVS was conducted under representative stomach conditions by incubating SYNB1618, without LAAD induction, in simulated gastric fluid (SGF) under 2% oxygen containing 20 mM Phe, and TCA production was determined by LC-MS/MS of SGF supernatants (*n* = 3 replicate incubations). Simulations of this IVS experimental design, performed using the SYNB1618 mechanistic model, accurately recapitulated TCA production over two hours (Fig. 1d), indicating that this mathematical representation adequately describes SYNB1618 PAL activity under simulated gastric conditions.

### Modeling SYNB1618 activity in the upper GI

**Fig. 2:**
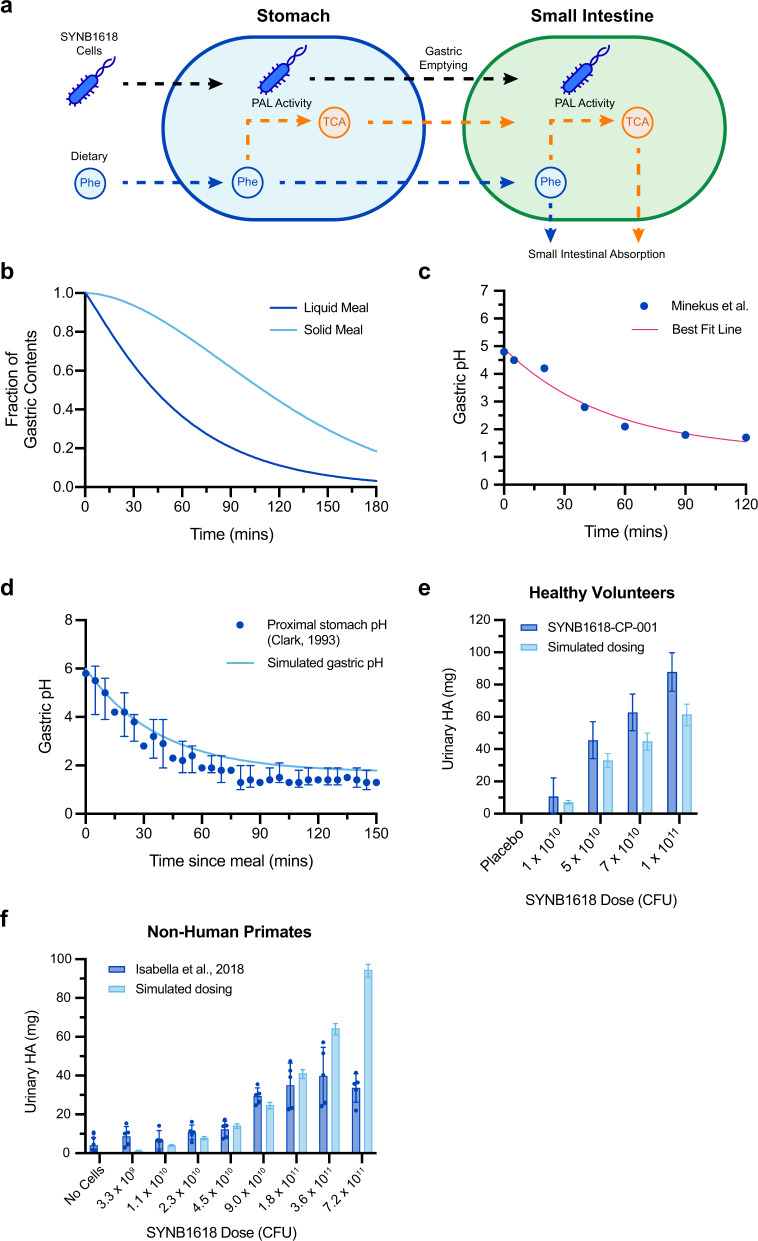
Mechanistic model of SYNB1618 PAL activity in the human upper GI. **a** Schematic representation of the human upper gastrointestinal SYNB1618 PAL activity model. The model is comprised of two compartments, with the stomach compartment emptying into the small intestine compartment. Conversion of Phe to TCA by SYNB1618 PAL in the stomach and small intestine are simulated using Michaelis-Menten kinetics. Absorption of Phe and TCA is also modeled in the small intestine. **b** Example of simulated gastric emptying curves using representative parameters for a liquid meal (*β* = 1.12, *t*_1/2_ = 43 min) or a solid meal (*β* = 1.81, *t*_1/2_ = 110 min). All simulations described in the main text assume liquid meals. **c** Postprandial gastric pH values recommended by Minekus et al.^36^ for in vitro simulation studies (blue) and best fit exponential decay curve employed for human upper gastrointestinal model (pink; *R*^2^ = 0.9677). **d** Simulated postprandial gastric pH (light blue) compared to proximal gastric pH of healthy subjects from Clark et al.^20^, following an oatmeal breakfast (median with interquartile ranges for *n* = 20 healthy volunteers; blue). **e** Urinary HA recovery (normalized to placebo and baseline values) from an SYNB1618 dose-escalation study in healthy subjects (blue; *n* = 8 subjects per dose level; mean ±SEM)^16^, compared to simulated SYNB1618 HA production using the human upper gastrointestinal activity model (light blue). **f**. Urinary HA recovery from NHP after administration of oral doses of SYNB1618 (blue; *n* = 5 animals per SYNB1618 dose level, *n* = 10 animals for no cells control; mean ±SEM)^14^, compared to simulated SYNB1618 HA production using parameters representing the NHP upper gastrointestinal tract (light blue). For **e**–**f** simulated data error bars represent 95% confidence intervals of the SYNB1618 PAL kinetic parameters, *K*m_PAL_ and *V*max_PAL_, as described in the main text.

We next sought to adapt the SYNB1618 PAL activity model to consider environmental conditions encountered by orally administered SYNB1618 in the human GI. As the small intestine is the primary site of Phe absorption in humans^17^, we focused our efforts on modeling SYNB1618 activity in the upper GI (stomach and small intestine). For the synthetic biotic, upper gastrointestinal transit represents a dynamic process comprised of gastric emptying^18^ and small intestinal motility^19^, changing pH^20–22^, decreasing oxygen^23,24^, and absorption of compounds by host tissues^17,25^. We implemented a two-compartment model representing SYNB1618 transit through the human stomach and small intestine that captured these various aspects of gastrointestinal physiology (Fig. 2a). Gastric emptying was represented using a power exponential function that is modifiable by dietary composition (Fig. 2b)^18^. Gastric pH increases transiently after a meal (pH 4.0–6.0, depending on meal composition) and then decreases to a basal level (pH < 2.0)^21^. This was modeled as an exponential decay from the postprandial gastric pH and fit to gastric pH values recommended for in vitro simulations by Minekus et al.^26^ (Fig. 2c). This representation was able to describe the postprandial gastric pH response in healthy subjects fed an oatmeal breakfast by providing only the initial measured gastric pH (Fig. 2d)^20^. Absorption of Phe and TCA in the small intestine was modeled following first-order kinetics. See *Methods* for a detailed description of the SYNB1618 upper GI model, assumptions, and parameter estimates.

We next aimed to validate predictions of the SYNB1618 PAL in vivo activity model. In a first-in-human dose-escalation study, healthy subjects received increasing doses of orally administered SYNB1618 in 100 mL masking solution containing sodium bicarbonate for buffering of stomach pH, together with a meal replacement shake containing 20 g protein (equivalent to 1.017 g Phe) and 15 mg/kg of body weight of isotopically labeled Phe (d5-Phe)^16^. Urine was collected over a 6-hour period after dosing to determine the total recovery of HA and d5-HA, revealing a dose-dependent production of these strain-specific biomarkers^16^. This clinical study design was simulated using the mechanistic model of SYNB1618 in vivo PAL activity for a single ascending dose of SYNB1618 in a 100 mL sodium bicarbonate solution, assuming co-administration of 2.0 g Phe and a total simulation time of 6 hours; corresponding to the period of observation in healthy subjects. Total TCA produced by SYNB1618 in these simulations was converted to HA, assuming 1:1 stoichiometry and complete recovery in urine^14^. Uncertainty in model predictions was incorporated using 95% confidence intervals for the estimates of the PAL activity parameters, *K*m_PAL_ and *V*max_PAL_. These simulations revealed an agreement with urinary HA data from healthy subjects dosed with SYNB1618 (Fig. 2e). Notably, the mechanistic model underestimated mean total HA recovery by ~30% across all simulated dose levels. There are several factors that may contribute to this discrepancy. For example, this may be attributable to consumption of endogenous Phe by SYNB1618 within the GI lumen of healthy subjects, made available by enterorecirculation^27^; that is not modeled. Alternatively, although we have assumed that TCA is absorbed by the human upper GI tract at the same rate as Phe, the bioavailability or rate of absorption of these compounds may differ.

To further demonstrate the translational value of this upper GI modeling framework, as well as to increase our confidence regarding estimates of SYNB1618 PAL activity in vivo, we also sought to validate model predictions using a preclinical animal model of SYNB1618 administration. In a previously published report, groups of NHP received increasing doses of orally administered SYNB1618 in a solution containing sodium bicarbonate for buffering of stomach pH, together with a bolus of peptone containing 5 g protein (equivalent to 0.25 g Phe)^14^. Notably, these NHP were administered SYNB1618 at doses that exceed those used for healthy human subjects. Urine was collected over a 6-hour period after dosing and revealed a dose-dependent production of urinary HA. This NHP study design was simulated using the mechanistic model of SYNB1618 in vivo PAL activity, and a set of parameters was selected to represent the upper GI of NHP. Total TCA produced by SYNB1618 in these simulations was again converted to HA, assuming 1:1 stoichiometry and complete recovery in urine. These simulations demonstrated agreement with urinary HA data from NHP dosed with SYNB1618 for doses up to 1.8 × 10^11^ colony-forming units (CFU) (Fig. 2f). However, simulated HA production exceeded HA levels recovered experimentally for the highest dose levels (3.6 × 10^11^ CFU and 7.2 × 10^11^ CFU). One interpretation of this discrepancy is that, at high dose levels, urinary HA secretion is limited by host metabolism (e.g., the bioavailability of intestinal TCA or the conversion of TCA to HA by host enzymes). Importantly, simulated and measured HA recovery were in close agreement at dose levels comparable to those used in healthy subjects and PKU patients. Taken together, these data support the utility of this model-based approach for estimating the activity of a synthetic biotic in the upper GI.

### Modeling Phe reduction in PKU patients

**Fig. 3:**
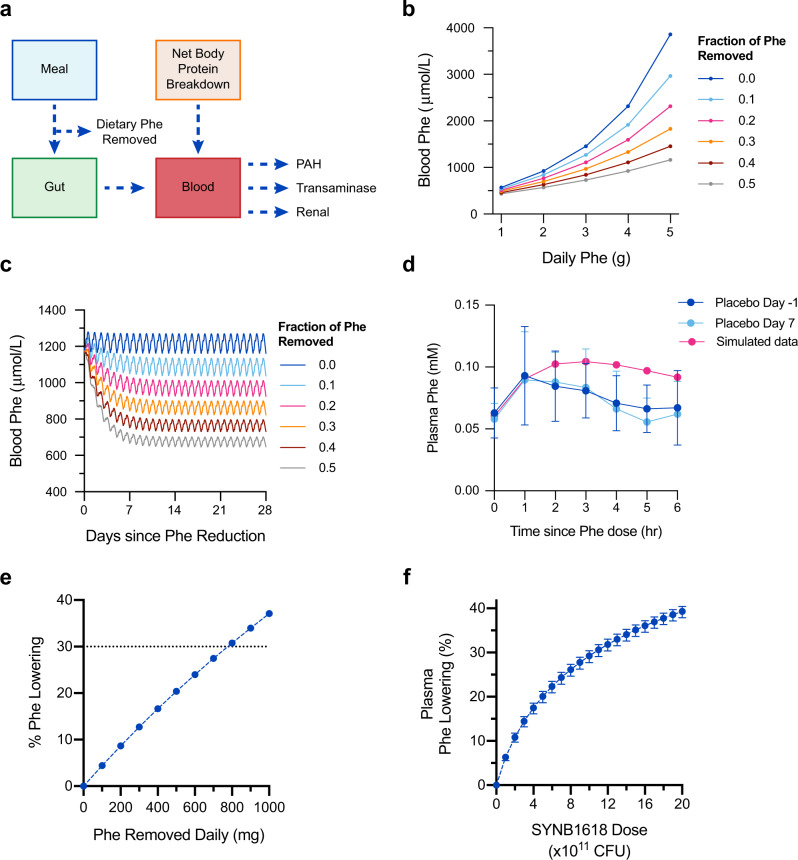
Model of blood Phe metabolism and blood Phe lowering with Phe removal from diet. **a** Schematic representation of blood Phe metabolism model, including contributions of Phe from meals and body protein breakdown, and elimination of Phe by PAH and transaminase activity, as well as renal excretion. Dietary Phe can be removed through SYNB1618 PAL activity. **b** Simulated fasting blood Phe concentrations for a classical PKU patient (0% PAH activity) across a range of dietary Phe intake and the effect of removal of 10–50% of dietary Phe on blood Phe concentrations. 180-day simulations were performed, and blood Phe concentrations 14 h following a meal are displayed. **c** Simulated blood Phe concentrations for a classical PKU patient (0% PAH activity) consuming 2.5 g Phe per day over 28 days, with the removal of 10–50% dietary Phe. **d** Simulated blood Phe concentrations in healthy subjects (100% PAH activity) receiving 1.0 g dietary Phe, compared with healthy subjects enrolled in the placebo arm of the first-in-human SYNB1618 clinical trial on study days ‒1 and 7 (*n* = 8 subjects per dose level; mean ±SD). **e** Estimated blood Phe lowering (%) for a classical PKU patient (0% PAH activity) following a nonadherent diet (2.5 g Phe daily), assuming 28 days of SYNB1618 dosing that consumes up to 1000 mg Phe daily. **f** Estimated blood Phe lowering (%) for a classical PKU patient (0% PAH activity) following a nonadherent diet (2.5 g Phe daily), assuming 28 days of SYNB1618 administration three-times daily with meals, using doses ranging from 0 to 2 × 10^12^ CFU, considering PAL activity only. Upper and lower bounds of simulated data represent 95% confidence intervals of the SYNB1618 PAL kinetic parameters, *K*m_PAL_ and *V*max_PAL_, as described in the main text.

Blood Phe lowering is the target of therapeutics for the treatment of PKU^28^. For example, clinical response to sapropterin dihydrochloride, an orally administered PAH enzyme cofactor analog that benefits a segment of PKU patients, is defined as a 30% decrease in blood Phe levels with treatment^12^. However, blood Phe levels in healthy subjects are under tight physiological regulation, limiting the feasibility of assessing the Phe-lowering capacity of any treatment in studies of healthy volunteers. To evaluate the effect of SYNB1618 PAL activity in the GI lumen on blood Phe levels, we developed a mechanistic model describing the relationship between dietary Phe and blood Phe concentrations (Fig. 3a). In brief, a previously published model of Phe metabolism in healthy subjects and PKU patients^29,30^ was extended to include the absorption of dietary Phe and renal Phe elimination (see *Methods* for a detailed description of model construction). Simulations of a diet comprised of 50 g of daily protein intake, corresponding to 2.5 g dietary Phe, result in steady-state blood Phe concentrations that align with clinical expectations. Specifically, simulations of patients with classical PKU (0, 1, and 2% of normal PAH activity) result in 1180, 860, and 660 µmol/L blood Phe concentration, whereas simulated heterozygote and healthy subjects have 96 and 65 µmol/L blood Phe, respectively. For classical PKU patients with 0% PAH activity, reducing dietary Phe intake by 20%, 30%, and 50% was estimated to decrease blood Phe levels by 21%, 30%, and 45%, respectively (Fig. 3b–c). Blood Phe levels approach steady-state ~5 days after changes to dietary Phe intake (Fig. 3c).

The addition of gut Phe absorption to this model was validated by comparison of simulations to postprandial Phe concentration data from healthy subjects enrolled in the placebo arm of the first-in-human SYNB1618 clinical study, who received a 100 mL meal replacement shake containing 20 g protein (equivalent to 1.017 g Phe) on two separate occasions (days −1 and 7 of the trial)^16^. Blood Phe kinetics were simulated by assuming oral administration of 1.0 g Phe, 100% PAH activity, and 0.06 mM baseline plasma Phe (equivalent to the initial values measured in these healthy subjects). The resulting simulations describe Phe absorption well (Fig. 3d). However, Phe elimination appears to return to the baseline concentration somewhat faster in healthy subjects than predicted by the model.

The extended blood Phe model was then used to determine the required Phe consumption by a synthetic biotic to lower blood Phe levels in PKU patients. Simulating dietary Phe removal in classical PKU patients on a 50 g daily protein diet (thus, assuming 0% PAH activity and 2.5 g dietary Phe daily), revealed that a synthetic biotic must consume ~780 mg per day of dietary Phe to result in a 30% lowering of blood Phe (Fig. 3e).

Finally, we combined these two mechanistic modeling approaches to estimate the dose of SYNB1618, considering PAL activity only, required to achieve a 30% blood Phe lowering in PKU patients. As comparable urinary HA levels were observed in healthy subjects and PKU patients dosed with SYNB1618^16^, validation of the PAL activity model using data from healthy subjects supports the use of this model for estimating SYNB1618 PAL activity in PKU patients. First, SYNB1618 dosing simulations were performed using the human upper gastrointestinal SYNB1618 PAL activity model and doses ranging from 1 × 10^11^ to 2 × 10^12^ CFU. Uncertainty in predicted SYNB1618 PAL activity was incorporated using 95% confidence intervals for the estimates of the PAL activity parameters, *K*m_PAL_ and *V*max_PAL_. The resulting SYNB1618 Phe consumption estimates by PAL were then removed from dietary Phe intake simulations using the extended blood Phe model, assuming patients with classical PKU (0% PAH activity) on a 50 g daily protein diet (corresponding to 2.5 g Phe/day) and three-times daily dosing of SYNB1618 with meals for 28 days. In all, 28-day simulations were performed to provide sufficient time to ensure pseudo-steady-state (intra-day variation due to meals, but no day-to-day variation) was reached subsequent to diet modification. These simulations revealed that the effect on blood Phe is estimated to range from 6.32% lowering with three-times daily dosing of 1 × 10^11^ CFU to 39.3% lowering at the 2 × 10^12^ CFU three-times daily dose level (Fig. 3f).

It is important to note that these simulations model the activity of the PAL pathway for Phe consumption only and do not consider Phe consumption by the LAAD enzyme expressed by SYNB1618. Given clinical evidence of LAAD activity in healthy volunteers^16^, these predictions of Phe lowering are thus likely to be underestimated, though the magnitude of the effect of LAAD on Phe lowering is uncertain. Nevertheless, these results suggest that an orally administered, Phe-consuming synthetic biotic can achieve reductions of plasma Phe in PKU patients.

## Discussion

In this article, we describe the development of mechanistic models designed to anticipate the activity of a synthetic biotic for the treatment of PKU, SYNB1618, in the human upper GI. The models described were able to accurately capture the activity of SYNB1618’s PAL pathway both in the context of IVS and in a clinical dose-escalation study with healthy subjects. Importantly, no significant difference in urinary HA levels was observed when comparing healthy volunteers to PKU patients dosed with SYNB1618^16^, suggesting that our simulated HA estimates are appropriate for both populations. Extension of a previously published blood Phe metabolism model was then used to estimate the effects of dietary Phe removal, as well as SYNB1618 PAL activity, on blood Phe lowering in PKU patients. These simulations suggest that it is feasible to achieve >20% blood Phe lowering in PKU patients with an orally administered synthetic biotic medicine at doses that are safe and well-tolerated in healthy subjects and patients^16^. The offset of Phe elimination between simulated and clinical plasma Phe data (Fig. 3d) suggests that future research could be extended to fit the clinical data and improve the model for predictivity in the clinic for healthy subjects and patients with PKU. Taken together, these observations indicate that mechanistic modeling approaches integrating in vitro simulation data with the knowledge of human gastrointestinal physiology can be used to predict the activity of an orally administered engineered bacterial therapeutic in humans.

However, there are limitations to this mechanistic modeling approach. First, all of the models implemented in this work are deterministic and may not capture the inherent variability present in the gastrointestinal function of heterogeneous human populations. Future implementations could incorporate this variability by stochastic sampling of upper gastrointestinal parameters (including pH, gastric emptying rate, and small intestinal absorption rates) from distributions that represent the relevant patient populations. Another limitation of this approach is that the dose of SYNB1618 is represented as a constant. Although SYNB1618 harbors an auxotrophy for diaminopimelic acid and rapidly clears from dosed subjects^16^, some degree of replication may occur in vivo. Similarly, although SYNB1618 was administered to healthy subjects and patients together with neutralizing buffer and a proton pump inhibitor^16^, it is also possible that a fraction of the SYNB1618 dose is lost owing to low pH in the stomach. These models could be extended to consider variability with respect to synthetic biotic viability in vivo. Finally, these models do not consider challenges presented by patient adherence. Notably, adherence to low-Phe PKU diets among adult patients is suboptimal^10^, indicating that a frequent dosing paradigm may also pose a challenge. As such, the clinical development of synthetic biotics must consider both the function of engineered strains in vivo as well as the requirements and preferences of patients.

In addition to dietary sources of Phe, secretion of Phe from systemic circulation into the gut lumen has been reported previously^14,27^. This phenomenon, termed enterorecirculation, is not currently considered, and our simulations thus represent a lower bound of SYNB1618 PAL activity, based on dietary Phe only. Enterorecirculation could be incorporated in the future as a potential source of systemic Phe for synthetic biotics in the gut lumen, particularly in the fasted state. Importantly, validation of the SYNB1618 PAL in vivo activity model described here also relied on the presence of urinary HA, a quantitative biomarker of strain activity^14,16^. Such biomarkers may be unavailable for other mechanisms of action (e.g., LAAD) and therapeutic areas, hindering the validation of mechanistic models. One approach to this problem may be to reuse components of validated models to develop “indirectly validated” mechanistic models for new applications and to support engineered strain development. Alternatively, mathematical models of synthetic biotic activity in vivo could benefit from applying non-targeted, metabolomics-based analysis of plasma and fecal metabolites in treated patients to elucidate effects on the host and bacterial metabolism that are not captured by targeted, tracer-based methods.

This mechanistic modeling approach is driven by parameter estimates obtained from in vitro studies with engineered bacterial strains. In vitro simulations in pure bacterial culture may be unable to capture all aspects of strain function in vivo, particularly if the therapeutic mechanism of action requires interaction with host tissues. However, recent advances in gut-on-a-chip technology enable the study of the interactions between bacteria and host tissues in microfluidic devices that emulate key aspects of human gastrointestinal physiology, including barrier function, mucus secretion, and peristaltic stretch^31–33^. In addition, these model systems can incorporate host immune cell populations and the endogenous human microbiota^34^. Moreover, independent microfluidic organ-on-a-chip models can be integrated in order to simulate the interaction between body systems^35^. These more sophisticated in vitro models present an opportunity to determine parameters that are inaccessible in pure culture, as well as to advance mechanistic models of synthetic biotic function.

In conclusion, synthetic biotics represent a category of living medicines that can be programmed to address mechanisms of disease within the body. Although synthetic biology techniques enable rapid design and construction of engineered strain prototypes, the development of any drug candidate requires rigorously designed, costly clinical studies to demonstrate safety and efficacy and to obtain regulatory approval^1^. Methods that leverage in vitro simulations and prior knowledge of human physiology to predict the impact of synthetic biotics on human disease can reduce the need for extensive preclinical animal studies and enable the selection of candidates with a high probability of success in clinical trials. Although animal models remain a critical component of preclinical research, the translational value of animal models is often limited, and a mechanistic modeling approach has the added benefit of reducing animal study requirements for evaluating strain function. As such, animal studies can be focused on answering key questions for which in vitro assays are not suitable. The models described here provide a proof-of-concept framework for applying mechanistic modeling techniques early in the development process to rapidly, confidently, and cost-effectively advance synthetic biotic candidates into clinical studies to address the unmet medical need of patients.

## Methods

### In vitro PAL parameter estimation studies

In vitro PAL parameter estimation studies were conducted by resuspending frozen aliquots of SYNB1618 in phosphate-buffered saline, pH 7.2 (PBS; ThermoFisher Scientific #20012027) at a concentration of 2.5 × 10^7^ CFU/mL. SYNB1618 PAL activity kinetic parameters, *K*m_PAL_ and *V*max_PAL_, were estimated by incubating cells with various concentrations of Phe (0.312–40.0 mM; Millipore Sigma #P2126) in triplicate and collecting cell-free supernatants after 60-mins incubation for quantitation of TCA by LC-MS/MS. Effects of pH on SYNB1618 PAL activity were estimated by incubating cells with 20 mM Phe in PBS calibrated to various pH levels (4.0–7.0) in triplicate. Cell-free supernatants were collected after 60-mins incubation for quantitation of TCA by LC-MS/MS. Inhibition of SYNB1618 PAL activity by exogenous TCA was determined by incubating cells with 20 mM d5-Phe (Cambridge Isotope Laboratories DLM-1258-PK) and increasing concentrations of unlabeled TCA (0.0–8.0 mM; Millipore Sigma #C80857). Cell-free supernatants were collected after 60-min incubation for quantitation of d5-TCA by LC-MS/MS.

### In vitro gastric simulation (IVS) assays

To characterize the viability and metabolic activity of SYNB1618 under physiological conditions, an IVS model was designed to simulate key aspects of oral administration in humans, including gastric oxygen concentration, pepsin secretion, and gastric pH. The IVS assay is comprised of incubations in 96-well microtiter plate format designed to simulate human stomach conditions (adapted from Minekus et al.^36^). In brief, frozen aliquots of SYNB1618 cells were first thawed at room temperature. Bacterial cell concentration is estimated by CFU plating or by counts of live and/or total cells (Cellometer K2 Image Cytometer; Nexelcom Bioscience). Aliquots of SYNB1618 are resuspended in 0.077 M sodium bicarbonate buffer at 5.0 × 10^9^ cells per mL. This solution is then mixed with equal parts of SGF^36^, containing 20 mM Phe, and incubated for 2 hours at 37°C with shaking in a polycarbonate in vitro hypoxic chamber (Coy Lab Products) calibrated to 2% oxygen. The resulting SYNB1618 cell density in SGF is 2.5 × 10^9^ cells/mL. To determine PAL activity, SGF aliquots were collected periodically and centrifuged at 4000 rpm for 5 mins using a tabletop centrifuge, followed by LC-MS/MS quantification of metabolites, including Phe and trans-cinnamate. Cell-free supernatants are optionally stored at −20°C until LC-MS/MS analysis.

### LC-MS/MS quantification of Phe and TCA

Quantification of analytes of interest was performed using a triple quadrupole LC-MS/MS Thermo TSQ Quantum Max system, using previously described methods^14,16^. In brief, 10 µL of plasma and diluted urine samples (1:40 with water) were transferred to a 96-well microtiter plate, followed by the addition of 90 µL derivatization solution (50 mM of 2-hydrazinoquinoline, dipyridyl disulfide, and triphenylphosphine in acetonitrile with 1 µg/mL of internal standard Phe-^13^C_9_-^15^N). The plate was incubated at 60°C for 1 h, and then centrifuged at 4000 rpm for 5 min. To another plate, 20 µL of the derivatized samples were transferred and further diluted with 180 μL of 0.1% formic acid in water/acetonitrile (140:40). The injection volume used was 10 µL and the run time was 4.25 min at a flow rate of 0.5 mL/min. Mobile phase A was 0.1% formic acid in water and mobile phase B was 0.1% formic acid in acetonitrile/isopropanol (90:10, v/v). Chromatographic separation was carried out using a Phenomenex Luna 5 µm C18 column (3 µm, 100 × 2 mm) with the following gradient: 10% B from 0 to 0.5 min, 10→97% B from 0.5 to 2 min, 97% B from 2 to 4 min, 10% B from 4 to 4.25 min. Multiple reaction monitoring (MRM) in positive mode was used for tandem MS analysis. The following mass transitions were monitored for quantitation: Phe (307.2/186.0), Phe-d_5_ (312.2/186.0), TCA (290.2/131.0), TCA-d_5_ (295.1/138.1), and internal standard Phe-^13^C_9_-^15^N (317.0/186.1).

### SYNB1618 PAL activity model

SYNB1618 TCA production by PAL was modeled as a differential equation assuming Michaelis–Menten enzyme kinetics, with each SYNB1618 cell representing the equivalent of one enzyme (Eqs. 1–2). This reaction is a single-step, irreversible conversion of Phe to its product, TCA. Inhibition constants were included to model the effects of pH and TCA inhibition on PAL activity (Eqs. 3–4). Parameters for Eqs. 1–4 were fit to in vitro experimental data of strain activity under the corresponding conditions (Table 1, Fig. 1a–c) using Prism 8.0 (GraphPad, San Diego, CA).

1 $$\frac{{{\rm{dPhe}}_{PAL}}}{{{\rm{d}}t}} =-\frac{{V{\rm{max}}_{\rm{PAL}}}}{{1+\frac{{K{\rm{m}}_{\rm{PAL}}}}{{\rm{Phe}}}}}\times {\rm{Cells}}\times K{\rm{i}}_{\rm{pH}}\times K{\rm{i}}_{{\rm{TCA}}}$$

2 $$\frac{{{\rm{dTCA}}_{PAL}}}{{\rm{d}}t} =\frac{{V{\rm{max}}_{\rm{PAL}}}}{1+\frac{{K{{\rm{m}}_{\rm{PAL}}}}}{{\rm{Phe}}}}\times {\rm{Cells}}\times K{\rm{i}}_{\rm{pH}}\times K{\rm{i}}_{\rm{TCA}}$$

3 $$K{\rm{i}}_{\rm{pH}}=0.25\times {\rm{pH}}-0.7$$

4 $$K{\rm{i}}_{\rm{TCA}}=0.67{e}^{-0.53\times {\rm{TCA}}}+0.33$$

**Table 1:** SYNB1618 PAL activity model parameters.

Parameter/state variable	Description	Value (Range)	Initial Value	Units
Phe	Phe concentration	-	40	μmol/mL
TCA	TCA concentration	-	0	μmol/mL
*t*	Time	-	-	min
Cells	SYNB1618 cell density	-	-	CFU/mL
*K*m_PAL_	Michaelis–Menten constant for SYNB1618 with Phe	0.0184 (0.005–0.0442)	-	μmol/mL
*V*max_PAL_	Maximum rate of TCA production by SYNB1618 PAL activity	0.0162 (0.0154–0.0171)	-	μmol/(min*10^9^ CFU)
*K*i_pH_	Inhibition constant for PAL due to pH	(0.0–1.0)	-	Unitless
*K*i_TCA_	Inhibition constant for PAL due to feedback inhibition by TCA	(0.0–1.0)	-	Unitless
pH	pH	(3.0–8.0)	-	Unitless

### Modeling SYNB1618 Activity in the Upper GI Tract

The human upper GI tract was modeled as a two-compartment system, representing the stomach and small intestine. Human gastrointestinal physiology, including gastric emptying, postprandial gastric pH, and metabolite absorption across the gut wall was modeled using data from the literature. Gastric emptying was modeled with a power exponential function, derived from Elashoff et al.^18^ (Eq. 5), that can represent the gastric emptying in response to both liquid and solid meals^18^. Postprandial gastric pH lowering was modeled as an exponential decay from the initial post-meal pH (Eq. 6). Parameters for this function (baseline pH and rate of decay) were fit to recommendations for IVS, as described by Minekus et al.^6^ (Fig. 2c), using Prism 8.0 (GraphPad, San Diego, CA). Absorption of Phe in the small intestine compartment was modeled using first-order kinetics, with an absorption constant for Phe derived from Adibi et al.^25^ (Eq. 7). Data were not available for the absorption of TCA, so the TCA absorption rate was assumed to be identical to that of Phe (Eq. 8). Due to the short duration of simulations (6 h), small intestinal emptying was not modeled.

5 $$f(t)={2}^{{-{(\frac{t}{{t_{{\frac{1}{2}}}}})}^{\beta }}}$$

6 $${\rm{pH}}_{{\rm{st}}}(t) = ({\rm{pH}}_{0}-{\rm{pH}}_{{{\rm{St}}\_{{\rm{min}}}}})\times {e}^{{-{k_{\rm{pH}}}t}}+{\rm{pH}}_{{{\rm{St}}\_{\rm{min}}}}$$

7 $${r}_{\rm{Phe,Abs}}=-\lambda \times {\rm{Phe}}_{\rm{SI}}$$

8 $${r}_{\rm{TCA,Abs}}=-\lambda \times {\rm{TCA}}_{\rm{SI}}$$

Following the function for gastric emptying (Eq. 5), changes in the number of SYNB1618 cells in the stomach and small intestine after dosing were expressed using Eqs. 9–10. Changes in Phe and TCA concentrations over time in the human upper GI tract with SYNB1618 dosing were represented by combining Eqs. 1–4 with Eqs. 5–8 to obtain Eqs. 11–12 for the stomach compartment and Eqs. 13–14 for the small intestine compartment. A schematic of the upper GI tract model is displayed in Fig. 2a. Simulations were performed by defining an initial state, including the dose of SYNB1618 and dietary Phe in the stomach compartment, and solving the system of ODEs numerically over a time period equal to 6 hours (i.e., the time interval for urine collections from healthy subjects and PKU patients after SYNB1618 dosing in the SYNB1618-CP-001 study)^16^. See Table 2 for a list of human upper gastrointestinal parameters and references. This model framework was also used to simulate SYNB1618 dosing in cynomolgus monkeys (NHP). See Table 3 for a list of NHP upper gastrointestinal parameters and references.

9 $$\frac{{\rm{dCells}}_{St}}{{\rm{d}}t}=-\left(\frac{{{\rm{df}}(t)}}{{{\rm{d}}t}}\times {\rm{Cells}}_{{St}}\right)$$

10 $$\frac{{{\rm{dCells}}_{SI}}}{{{\rm{d}}t}} = \left(\frac{{{\rm{df}}(t)}}{{{\rm{d}}t}}\times {\rm{Cells}}_{{St}}\right)$$

11 $$\frac{{{\rm{dPhe}}_{St}}}{{{\rm{d}}t}}=-\left(\frac{{V{\rm{max}}_{\rm{PAL}}}}{{1+\frac{{K{{\rm{m}}_{\rm{PAL}}}}}{{{\rm{Phe}}_{\rm{St}}}}}}\times {\rm{Cells}}_{St}\times K{\rm{i}}_{{{\rm{pH}}_{\rm{St}}}}\times K{\rm{i}}_{{{\rm{TCA}}_{\rm{St}}}}\right)-\left(\frac{{{\rm{df}}t}}{{{\rm{d}}t}}\times {\rm{Phe}}_{\rm{St}}\right)$$

12 $$\frac{{{\rm{dTCA}}_{St}}}{{{\rm{d}}t}}=\left(\frac{{V{\rm{max}}_{\rm{PAL}}}}{{1+\frac{{K{{\rm{m}}_{\rm{PAL}}}}}{{{\rm{Phe}}_{\rm{St}}}}}}\times {\rm{Cells}}_{St}\times K{\rm{i}}_{{{\rm{pH}}_{\rm{St}}}}\times K{\rm{i}}_{{{\rm{TCA}}_{\rm{St}}}}\right)-\left(\frac{{{\rm{df}}t}}{{{\rm{d}}t}}\times {\rm{TCA}}_{\rm{St}}\right)$$

13 $$\frac{{{\rm{dPhe}}_{SI}}}{{{\rm{d}}t}}=-\left(\frac{{V{\rm{max}}_{\rm{PAL}}}}{{1+\frac{{K{{\rm{m}}_{\rm{PAL}}}}}{{{\rm{Phe}}_{SI}}}}}\times {\rm{Cells}}_{\mathrm{SI}}\times K{\rm{i}}_{{{\rm{pH}}_{\rm{SI}}}}\times K{\rm{i}}_{{{\rm{TCA}}_{\rm{SI}}}}\right)+\left(\frac{{{\rm{df}}(t)}}{{{\rm{d}}t}}\times {\rm{Phe}}_{\rm{St}}\right)-{r}_{\rm{Phe,Abs}}$$

14 $$\frac{{{\rm{dTCA}}_{SI}}}{{{\rm{d}}t}}=\left(\frac{{V{\rm{max}}_{\rm{PAL}}}}{{1+\frac{{K{{\rm{m}}_{\rm{PAL}}}}}{{{\rm{Phe}}_{SI}}}}}\times {\rm{Cells}}_{\rm{SI}}\times K{\rm{i}}_{{{\rm{pH}}_{SI}}}\times K{\rm{i}}_{{{\rm{TCA}}_{SI}}}\right)+\left(\frac{{{\rm{df}}(t)}}{{{\rm{d}}t}}\times {\rm{TCA}}_{\rm{St}}\right)-{r}_{\rm{TCA,Abs}}$$

**Table 2:** Human upper gastrointestinal model parameters.

Parameter/state variable	Description	Value (range)	Initial value	Units	Source
*t*	Time	-	-	min	
f	Fraction of stomach contents remaining (relative to initial state)	(0.0–1.0)	1	Unitless	Elashoff 1982^18^
$\beta$	Gastric emptying curve shape parameter	1.12	-	Unitless	Elashoff 1982^18^
*t*_1/2_	Half gastric emptying time	43.0	-	min	Elashoff 1982^18^
Phe_St_	Stomach Phe content	-	12108	μmol	
TCA_St_	Stomach TCA content	-	0	μmol	
Cells_St_	Stomach SYNB1618 content	-	Dose-dependent	Cells	
pH_St_	Stomach pH	(1.71–8.0)	-	Unitless	
pH_0_	Initial stomach pH	6.0	-	Unitless	Simmons 1986^21^
pH_St_min_	Minimum gastric pH	1.71	-	Unitless	Minekus 1995^26^
*k*_pH_	Gastric pH decay constant	0.02653	-	min^−1^	Minekus 1995^26^
Phe_SI_	Small intestinal Phe content	-	30.2	μmol	
TCA_SI_	Small intestinal TCA content	-	0	μmol	
Cells_SI_	Small intestinal SYNB1618 content	-	0	Cells	
pH_SI_	Small intestinal pH	6.5	-	Unitless	Minekus 2014^36^
Vol_St_	Fasted stomach volume	35.0	-	mL	Mudie 2014^39^
Vol_SI_	Small intestinal volume	54.0	-	mL	McConnell 2008^22^
$\lambda$	Small intestinal absorption rate	0.1019	-	min^−1^	Adibi 1967^25^

**Table 3:** Non-human primate upper gastrointestinal model parameters.

Parameter/state variable	Description	Value (range)	Initial value	Units	Source
t	Time	-	-	min	
*f*	Fraction of stomach contents remaining (relative to initial state)	(0.0–1.0)	1	Unitless	
$\beta$	Gastric emptying curve shape parameter	1.12	-	Unitless	Human parameter
*t*_1/2_	Half gastric emptying time	24.5	-	min	Kondo 2003a^40^
Phe_St_	Stomach Phe content	-	1513.41	μmol	Isabella 2018^14^
TCA_St_	Stomach TCA content	-	0	μmol	
Cells_St_	Stomach SYNB1618 content	-	Dose-dependent	Cells	
pH_St_	stomach pH	(1.97–8.0)	-	Unitless	Chen 2008^41^, Kondo 2003b^42^
pH_0_	Initial stomach pH	6.0	-	Unitless	Chen 2008^41^, Kondo 2003b^42^
pH_St_min_	Minimum gastric pH	1.97	-	Unitless	Kondo 2003b^42^
*k*_pH_	Gastric pH decay constant	0.004	-	min^−1^	Kondo 2003b^42^
Phe_SI_	Small intestinal Phe content	-	7.7	μmol	Estimated from SI volume
TCA_SI_	Small intestinal TCA content	-	0	μmol	
Cells_SI_	Small intestinal SYNB1618 content	-	0	Cells	
pH_SI_	Small intestinal pH	5.8	-	Unitless	Hatton 2015^4^
Vol_St_	Fasted stomach volume	17.0	-	mL	Unpublished NHP study
Vol_SI_	Small intestinal volume	13.7	-	mL	Unpublished NHP study
$\lambda$	Small intestinal absorption rate	0.1019	-	min^−1^	Human parameter

### Extension of Phe metabolism model to incorporate dietary Phe intake

A published model of Phe concentration-time profiles with varying levels of PAH function^29,30^ was implemented, with errata corrections, and expanded to describe meal effects. The model was then used to simulate the effect of varying degrees of meal Phe reduction on Phe concentrations over time. This model assumes two primary methods of Phe elimination in humans, PAH and transaminase enzyme activity (Eqs. 15–16), and was modified to include renal excretion of Phe (Eq. 17; estimated using data extracted from Kitagawa^37^). In addition, endogenous protein breakdown is the only source of blood Phe production in the original model^29^. The model was also extended to include Phe absorption from the gut, assuming complete Phe bioavailability and rapid absorption after the meal (Eq. 18). A unit conversion for gut Phe content (mg) to concentration in blood (mmol/L) was also performed (Eq. 19). The change of blood Phe concentrations in the extended model was thus represented using (Eq. 20). Simulations assumed 2.5 g Phe consumed per day, divided evenly into three meals, occurring at 0, 4, and 10 h of the day, relative to 8:00 AM. The equations are below. According to this model, in patients with classical PKU (0% PAH activity), PAH, transaminase, and renal elimination mechanisms are responsible for ~0%, 99%, and 1% of Phe elimination, respectively. See Table 4 for a list of blood Phe metabolism parameters.

15 $${V}_{\rm{PAH}}=\frac{{V}_{\rm{max,PAH}}\times {F}_{\rm{PAH}}}{1+\frac{{K}_{\rm{m,PAH}}}{\rm{Phe}}+\frac{{K}_{\rm{m,PAH}}\times {K}_{\rm{a,PAH}}}{{\rm{Phe}}^{2}}}$$

16 $${V}_{\rm{trans}}=\frac{{V}_{\rm{max,trans}}}{1+\frac{{K}_{\rm{m,trans}}}{\rm{Phe}}}$$

17 $${V}_{\rm{renal}}={\rm{Phe}}\times {\rm{CL}}_{\rm{renal}}\times {V}_{\rm{d}}$$

18 $$\frac{\rm{dGut}}{{\rm{d}}t}=-{K}_{\rm{a,gut}}\times {\rm{Gut}}$$

19 $${F}_{\rm{Gut,Plasma}}=\frac{1}{{\rm{MW}}_{\rm{Phe}}\times {V}_{d}\times W}$$

20 $$\frac{{\rm{dPhe}}}{{\rm{d}}t}={K}_{\rm{a,gut}}\times {\rm{Gut}}\times {F}_{\rm{Gut,Plasma}}+{V}_{\rm{npd}}-{V}_{\rm{PAH}}-{V}_{\rm{trans}}-{V}_{\rm{renal}}$$

**Table 4:** Blood Phe metabolism model parameters.

Parameter/state variable	Description	Value	Initial value	Units
Phe	Plasma Phe concentration	-	1.18	mmol/L
Gut	Gut Phe content	-	0	mg
*t*	Time	-	-	hr
*K*_a,gut_	Absorption rate from the gut to plasma	0.25	-	1/hr
*V*_npd_	Rate of net protein breakdown	0.012	-	(mmol/L)/hr
*V*_PAH_	Rate of Phe breakdown by PAH	-	-	(mmol/L)/hr
*V*_trans_	Rate of Phe breakdown by transaminase	-	-	(mmol/L)/hr
*V*_renal_	Rate of renal Phe elimination	-	-	(mmol/L)/hr
*V*_max,PAH_	Maximum rate of Phe breakdown by PAH in a healthy subject	0.9	-	(mmol/L)/hr
*F*_PAH_	Fraction of healthy PAH activity	0.0–1.0	-	Unitless
*K*_m,PAH_	Michaelis–Menten constant for Phe with PAH	0.51	-	mmol/L
*K*_a,PAH_	Phe activation constant for PAH	0.54	-	mmol/L
*V*_max,trans_	Maximum rate of Phe breakdown by transaminase	0.063	-	(mmol/L)/hr
*K*_m,trans_	Michaelis–Menten constant for Phe with transaminase	1.37	-	mmol/L
CL_renal_	Renal clearance of Phe per body weight	5.696 × 10^−4^	-	(L/kg)/hr
*F*_Gut,Plasma_	Unit conversion adjustment from Gut to Plasma concentrations	-	-	(mmol/L)/mg
MW_Phe_	Molecular weight of Phe	165.19	-	g/mol
*V*_d_	Volume distribution of Phe	0.5	-	L/kg
*W*	Body weight	70	-	kg

### Statistics and reproducibility

All in vitro assays were performed using *n* = 3 independent bacterial cultures per condition and the data presented are representative of two or more independent experiments. Several parameters for the SYNB1618 in vitro activity and human upper gastrointestinal model were fit to experimental data using Prism 8.0 (GraphPad, San Diego, CA). The goodness of fit of nonlinear or linear functions to experimental data was described by *R*^2^ value, as determined using Prism 8.0.

### Clinical study oversight

The SYNB1618-CP-001 study was sponsored by Synlogic Inc. (Cambridge, MA). The study received Institutional Review Board (IRB) approval and was registered on ClinTrials.gov (NCT03516487). All subjects enrolled in the study gave informed consent, and the study was conducted under Good Clinical Practice regulations and the Declaration of Helsinki. The study was conducted at four clinical research centers in the United States. A safety review committee reviewed safety data after each cohort completed dosing.

### Reporting summary

Further information on research design is available in the Nature Research Reporting Summary linked to this article.

## Supplementary information

Supplementary Information

Description of Supplementary Files

Supplementary Data 1

Reporting Summary

## Data Availability

Source data for all figures are available in tables available in Supplementary Data 1. Data sourced from Clark et al., 1993 (Fig. 2d) and the clinical trial SYNB1618-CP-001 (Figs. 2e and 3d) were published as summary data, and as such, individual data points are not available. Simulated data are shown as median values with confidence intervals, as these data are not experimentally measured replicates, but rather represent a range of simulated outputs based on confidence intervals on the parameters, as described in the main text.

## Code availability

The SYNB1618 in vitro activity model, the SYNB1618 human upper GI tract activity model, and the extended blood Phe metabolism model were all implemented in Python 3.7.4, using Jupyter version 6.0.1 (jupyter.org). Ordinary differential equations were solved using SciPy version 1.5.2 (scipy.org). Jupyter notebooks for the SYNB1618 in vitro activity model, the SYNB1618 human and NHP upper GI tract activity models, and the extended blood Phe metabolism model are available for download on zenodo.org^38^.
