# Supplementary material for: Development of a mechanistic model to predict synthetic biotic activity in healthy volunteers and patients with phenylketonuria
Source: Commun Biol. 2021 Jul 22;4:898. doi: 10.1038/s42003-021-02183-1 (PMC8298439; doi:10.1038/s42003-021-02183-1)
Supplement: Supplementary file 1 — Supplementary Information [file 42003_2021_2183_MOESM1_ESM.docx]

**Supplementary Information**

**Development of a Mechanistic Model to Predict Synthetic Biotic Activity in Healthy Volunteers and Patients with Phenylketonuria**

Mark R. Charbonneau^1*^, William S. Denney^2^, Nicholas Horvath^1^, Pasquale Cantarella^1^, Mary J. Castillo^1^, Marja K. Puurunen^1^, Aoife M. Brennan^1^

^1^Synlogic, Inc. 301 Binney Street, Cambridge, MA 02142, USA

^2^Human Predictions, LLC. Cambridge, MA 02139, USA

*Corresponding author: [mark@synlogictx.com](mailto:mark@synlogictx.com)


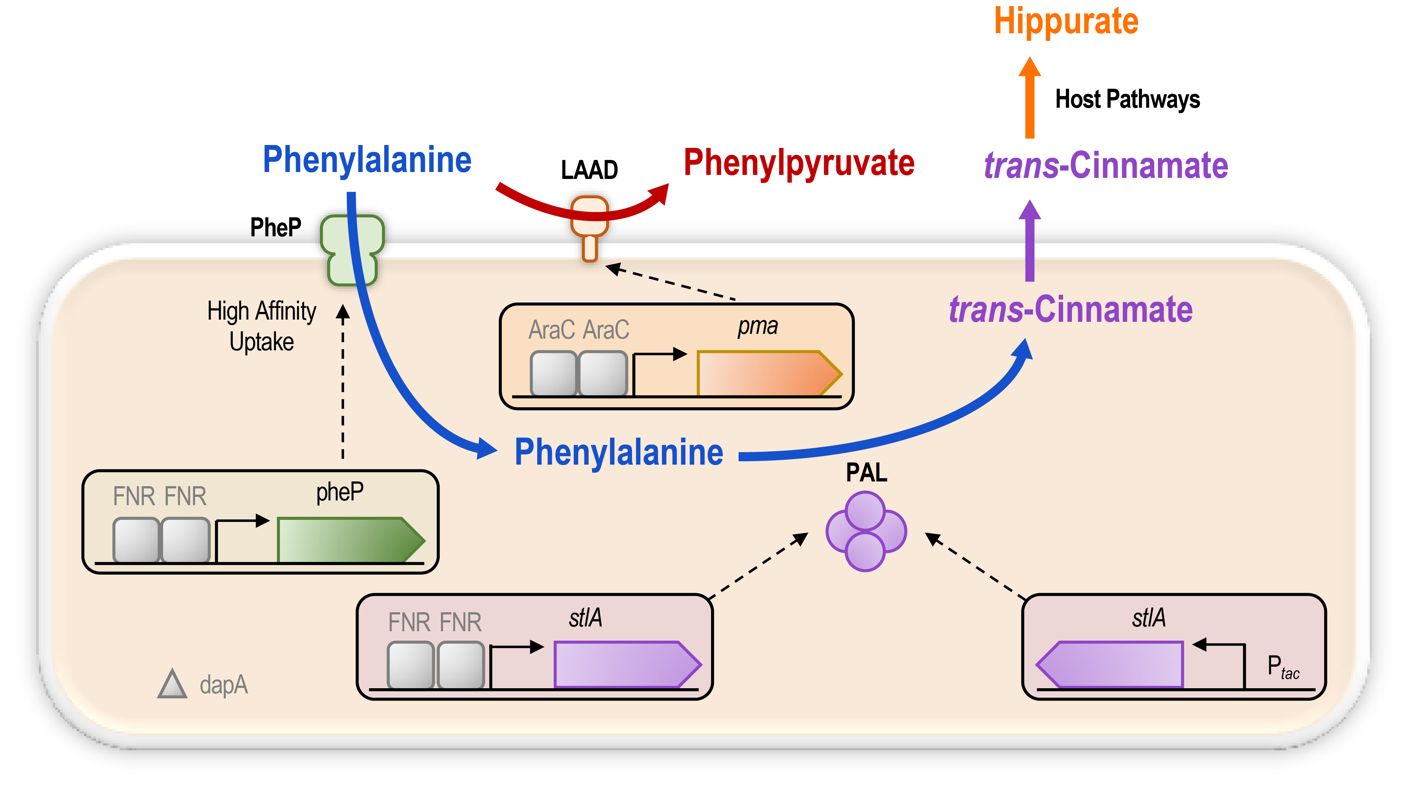


**Supplementary Figure 1. Design of SYNB1618 (Adapted from Isabella et al., 2018**^1^**).** SYNB1618 contains chromosomally inserted genes encoding PheP, a high affinity Phe transporter that can bring Phe into the cytoplasm, PAL, which converts Phe to TCA, and LAAD, which converts Phe to PPA. Induction of these components is carried out partially by the anaerobic-responsive transcriptional activator FNR, for strain activation of PAL and PheP in the anoxic environment of the mammalian gastrointestinal tract. Additional copies of PAL and LAAD are placed under control of the Isopropyl-beta-D-l-thiogalactopyranoside, and L-arabinose inducible promoters, respectively, for strain activation *in vitro* preparation of the drug product.

**Supplementary References**

1. Isabella, V. M. *et al.* Development of a synthetic live bacterial therapeutic for the human metabolic disease phenylketonuria. *Nat. Biotechnol.* **36**, 857–864 (2018).
