# Supplementary material for: Development of a mechanistic model to predict synthetic biotic activity in healthy volunteers and patients with phenylketonuria
Source: Commun Biol. 2021 Jul 22;4:898. doi: 10.1038/s42003-021-02183-1 (PMC8298439; doi:10.1038/s42003-021-02183-1)
Supplement: Supplementary file 2 — Description of Supplementary Files [file 42003_2021_2183_MOESM2_ESM.pdf]

Description of Additional Supplementary Files

File name: Supplementary Data

Description: Source data underlying all graphs and charts.
