# Supplementary material for: Development of a mechanistic model to predict synthetic biotic activity in healthy volunteers and patients with phenylketonuria
Source: Commun Biol. 2021 Jul 22;4:898. doi: 10.1038/s42003-021-02183-1 (PMC8298439; doi:10.1038/s42003-021-02183-1)
Supplement: Supplementary file 4 — Reporting Summary [file 42003_2021_2183_MOESM4_ESM.pdf]

nature research

Corresponding author(s): Mark R Charbonneau

Last updated by author(s): 21 Apr 2021

Reporting Summary

Nature Research wishes to improve the reproducibility of the work that we publish. This form provides structure for consistency and transparency in reporting. For further information on Nature Research policies, see our Editorial Policies and the Editorial Policy Checklist.

Statistics

For all statistical analyses, confirm that the following items are present in the figure legend, table legend, main text, or Methods section.

n/a	Confirmed	
☐	☒	The exact sample size (*n*) for each experimental group/condition, given as a discrete number and unit of measurement
☐	☒	A statement on whether measurements were taken from distinct samples or whether the same sample was measured repeatedly
☒	☐	The statistical test(s) used AND whether they are one- or two-sided<br>*Only common tests should be described solely by name; describe more complex techniques in the Methods section.*
☒	☐	A description of all covariates tested
☐	☒	A description of any assumptions or corrections, such as tests of normality and adjustment for multiple comparisons
☐	☒	A full description of the statistical parameters including central tendency (e.g. means) or other basic estimates (e.g. regression coefficient) AND variation (e.g. standard deviation) or associated estimates of uncertainty (e.g. confidence intervals)
☒	☐	For null hypothesis testing, the test statistic (e.g. *F*, *t*, *r*) with confidence intervals, effect sizes, degrees of freedom and *P* value noted<br>*Give P values as exact values whenever suitable.*
☒	☐	For Bayesian analysis, information on the choice of priors and Markov chain Monte Carlo settings
☒	☐	For hierarchical and complex designs, identification of the appropriate level for tests and full reporting of outcomes
☒	☐	Estimates of effect sizes (e.g. Cohen's *d*, Pearson's *r*), indicating how they were calculated

Our web collection on statistics for biologists contains articles on many of the points above.

Software and code

Policy information about availability of computer code

Data collection	Mechanistic models were all implemented in Python 3.7.4, using Jupyter version 6.0.1 (jupyter.org). Ordinary differential equations were solved using SciPy version 1.5.2 (scipy.org). LC-MS/MS analysis was performed using Thermo Xcalibur software, version 3.0.
Data analysis	Several parameters for the SYNB1618 in vitro activity and human upper gastrointestinal model were fit to experimental data using Prism 8.0 (GraphPad, San Diego, CA). The SYNB1618 in vitro activity model, the SYNB1618 human upper gastrointestinal tract activity model, and the extended blood Phe metabolism model were all implemented in Python 3.7.4, using Jupyter version 6.0.1 (jupyter.org). Ordinary differential equations were solved using SciPy version 1.5.2 (scipy.org).

For manuscripts utilizing custom algorithms or software that are central to the research but not yet described in published literature, software must be made available to editors and reviewers. We strongly encourage code deposition in a community repository (e.g. GitHub). See the Nature Research guidelines for submitting code & software for further information.

Data

Policy information about availability of data

All manuscripts must include a data availability statement. This statement should provide the following information, where applicable:

- Accession codes, unique identifiers, or web links for publicly available datasets
- A list of figures that have associated raw data
- A description of any restrictions on data availability

Jupyter notebooks for the SYNB1618 in vitro activity model, the SYNB1618 human and NHP upper gastrointestinal tract activity models, and the extended blood Phe metabolism model are available for download on zenodo.org (doi:10.5281/zenodo.4708073).

Field-specific reporting

Please select the one below that is the best fit for your research. If you are not sure, read the appropriate sections before making your selection.

- [x] Life sciences
- [ ] Behavioural & social sciences
- [ ] Ecological, evolutionary & environmental sciences

For a reference copy of the document with all sections, see nature.com/documents/nr-reporting-summary-flat.pdf

Life sciences study design

All studies must disclose on these points even when the disclosure is negative.

Sample size	Sample size calculations were not performed for in vitro studies. The sample sizes used throughout this study are sufficient due to the low variability present in the IVS and bioanalytical assays.
Data exclusions	No data were excluded.
Replication	All in vitro studies were replicated at least twice and demonstrated reproducible results.
Randomization	No randomization was performed. Since all assays performed involved bacterial monocultures, randomization was not required.
Blinding	Blinding of samples for in vitro assays was not performed. All assays utilized were quantitative and implemented internal standards and system suitability criteria to ensure data integrity.

Reporting for specific materials, systems and methods

We require information from authors about some types of materials, experimental systems and methods used in many studies. Here, indicate whether each material, system or method listed is relevant to your study. If you are not sure if a list item applies to your research, read the appropriate section before selecting a response.

Materials & experimental systems

n/a	Involved in the study	
x		Antibodies
x		Eukaryotic cell lines
x		Palaeontology and archaeology
x		Animals and other organisms
x		Human research participants
x		Clinical data
x		Dual use research of concern

Methods

n/a	Involved in the study	
x		ChIP-seq
x		Flow cytometry
x		MRI-based neuroimaging
